# Editorial: Host-microbiota immuno-interactions for personalized microbial therapeutics

**DOI:** 10.3389/fimmu.2025.1716098

**Published:** 2025-10-28

**Authors:** Shashank Gupta, Sunil Kumar Raghav, Nar Singh Chauhan

**Affiliations:** ^1^ School of Medical Sciences, Faculty of Medicine and Health, Örebro University, Örebro, Sweden; ^2^ Laboratory of Immuno-genomics & Systems Biology, Institute of Life Sciences, Bhubaneswar, Orissa, India; ^3^ Department of Biochemistry, Maharshi Dayanand University, Rohtak, Haryana, India

**Keywords:** human microbiota, host-microbe interactions, personalized therapy, microbiome therapeutics, microbiota dysbiosis

The human gut microbiota plays a crucial role in modulating the immune system by regulating both innate and adaptive immune responses (1). Dysbiosis of the gut microbiota can disrupt these immune-interactions and predispose individuals to immune-mediated physiological and metabolic disorders (2). Dysbiosis of the human gut microbiota often manifests as increased gut permeability, aberrant immune responses, and chronic inflammation (3). Restoring a balanced microbiota through microbial therapeutics has emerged as a promising strategy to mitigate these detrimental effects (4). Personalized microbial therapeutics are developed to exploit host-microbiota interactions in a manner tailored to an individual’s microbiome composition and immune profile (5). Recent developments in human microbiome research highlight the need for personalized therapeutic approaches that address the complex interplay between the immune system and the microbiota (6). This Research Topic brings together compelling articles that highlight diverse ways in which host-microbiota immune interactions can be leveraged to improve personalized microbial therapeutics.

## Research and review articles

Disease-focused clinical perspectives highlight the role of microbial communities in pathophysiology and patient outcomes. Belayneh et al. delineated the role of *Helicobacter pylori* co-infection in the alteration of clinical features in hepatitis B patients, providing evidence that microbial status can influence viral disease trajectories. Deng et al. connected alterations in the vaginal microbiota to preterm premature rupture of membranes as prognostic biomarkers, offering a pathway for early intervention in pregnancy complications. Guo et al. highlighted the gut–immune axis in primary immune thrombocytopenia, reframing autoimmune pathology through a microbiome lens and pointing toward novel treatment paradigms. Eslami et al. explored microbiota as diagnostic biomarkers, emphasizing their potential for early cancer detection and personalized treatment planning. Chen et al. demonstrated that *Pseudomonas aeruginosa* can enhance the effectiveness of anti-PD-1 therapy for colorectal cancer by activating CD8^+^ T cells, underscoring the potential for synergistic effects between microbiota and immunotherapy. Complementing these insights, Nieves et al. discussed how microbiome modulation could improve clinical outcomes in immunocompromised patients. Together, these findings highlight the promise of microbiota-informed diagnostics and therapeutic strategies in diverse clinical settings.

Microbial therapeutic innovations also provide a broader framework for personalized therapeutics. Nazir et al. summarized approaches to therapeutically target the host–microbiota–immune axis, emphasizing the potential of precision microbial medicine. Simultaneously, Ding et al. highlighted the role of the microbiome in combating antimicrobial resistance, projecting microbiota-based interventions as a promising frontier in addressing one of the greatest global challenges. Additionally, Wang et al. summarized microbial and metabolite targets that could be leveraged to develop personalized microbiota-based therapies for pediatric pulmonary diseases. Experimental studies enrich our clinical and translational understanding with insights into host–microbe biology. Li et al. showed the role of commensal bacterial history in calibrating antiviral immune readiness and influencing the threshold for adaptive immune activation. Liu et al. demonstrated the role of Chaihushugan powder in alleviating functional dyspepsia in rats by reshaping the gut microbiota and reducing mitochondrial oxidative stress in gastric tissue. These findings highlight the innovative approaches to harnessing the microbiota for therapeutic purposes.

This Research Topic charts a promising roadmap for translating microbiome science into precision therapeutics. The integration of mechanistic insights, translational studies, and clinical evidence provides a robust framework that advances microbiota-informed personalized medicine as a foundational paradigm in future healthcare (7).
